# The clinically-integrated randomized trial: proposed novel method for conducting large trials at low cost

**DOI:** 10.1186/1745-6215-10-14

**Published:** 2009-03-05

**Authors:** Andrew J Vickers, Peter T Scardino

**Affiliations:** 1 Department of Epidemiology and Biostatistics, Memorial Sloan Kettering Cancer Center, 1275 York Avenue, NY, NY 10021, USA

## Abstract

**Introduction:**

Randomized controlled trials provide the best method of determining which of two comparable treatments is preferable. Unfortunately, contemporary randomized trials have become increasingly expensive, complex and burdened by regulation, so much so that many trials are of doubtful feasibility.

**Discussion:**

Here we present a proposal for a novel, streamlined approach to randomized trials: the "clinically-integrated randomized trial". The key aspect of our methodology is that the clinical experience of the patient and doctor is virtually indistinguishable whether or not the patient is randomized, primarily because outcome data are obtained from routine clinical data, or from short, web-based questionnaires. Integration of a randomized trial into routine clinical practice also implies that there should be an attempt to randomize every patient, a corollary of which is that eligibility criteria are minimized. The similar clinical experience of patients on- and off-study also entails that the marginal cost of putting an additional patient on trial is negligible. We propose examples of how the clinically-integrated randomized trial might be applied in four distinct areas of medicine: comparisons of surgical techniques, "me too" drugs, rare diseases and lifestyle interventions. Barriers to implementing clinically-integrated randomized trials are discussed.

**Conclusion:**

The proposed clinically-integrated randomized trial may allow us to enlarge dramatically the number of clinical questions that can be addressed by randomization.

## Introduction

Consider that participation in a typical randomized trial is a quite distinct from usual clinical care. From the patients' point of view, participation in a trial generally requires additional tests, clinic visits and procedures such as scans and biopsies, as well as a large number of questionnaires. For the doctor, having a patient on trial involves a considerable amount of additional paperwork, everything from documenting eligibility, recording the results of protocol specific tests and fulfilling regulatory requirements: a patient on chemotherapy who had a moderately abnormal white count would be followed carefully; if the the same patient was on a chemotherapy trial, the abnormal white count would likely necessitate several letters to various oversight bodies describing the adverse event, the causal relationship to the investigational agent and the resulting medical treatment. Participation in a randomized trial is also made distinct by its rarity: a review of randomized trials in surgery, for example, estimated that typically fewer than 1% of eligible patients are accrued[1]. Indeed, there is a considerable literature on the difficulties of accruing patients to clinical trials[2,3].

## Discussion

In this paper, we develop a novel framework for thinking about and conducting randomized trials. We propose to integrate randomized trials and routine clinical practice, a design we term the "clinically-integrated randomized trial". The key principle is that the *clinical experience of the patient and doctor is virtually indistinguishable whether or not the patient is randomized*. Trial patients go through informed consent procedures, and certain aspects of care, such as modifications to the surgical technique used, are determined by randomization rather than being at the discretion of the doctor. Otherwise, there are no obvious differences between the clinical care, follow-up, payment and documentation requirements between patients who do and do not participate in the trial.

Integration of a randomized trial into routine clinical practice also implies that randomization itself is routine, in other words, *there should be an attempt to randomize every patient*. A corollary is that *eligibility criteria need to be minimized*. The only eligibility criterion should be that the doctor is uncertain about which of the treatments in the trial would be best for the patient, the "uncertainty principle"[4].

Ensuring that the clinical experience of patients on trial is similar to those off-study has an important financial implication: *the marginal cost of putting an additional patient on trial is negligible*. Once the study infrastructure (such as the study database) is established, and other expenses associated with trial initiation (such as steering a trial through scientific review) are accounted for, the only substantive cost incurred by accrual of a specific patient is the time involved to inform the patient of the trial and obtain consent. The costs of each additional randomization is trivial, especially if conducted by computer; patients then have to be treated, but this is true whether or not they take part in the trial. Under the assumption that a fixed proportion of patients will be audited, accrual of a patient will increase auditing costs, but only by a small amount. This is in sharp distinction to a traditional randomized trial, where the extra visits, tests and questionnaires incur substantial costs, particularly because staff must be hired to manage trial patients and protocol requirements.

Integration of a trial into routine clinical practice will generally only be possible if *patients, doctors and payers do not generally have a strong preference for one technique over another*. This would not be the case of trials comparing very different surgical approaches (e.g. laparoscopic versus open surgery), active treatment versus active surveillance, or a new procedure that incurs much higher costs than the traditional approach. Randomized trials of such comparisons are no doubt important, but will either include only a subgroup of patients without a strong preference for one or other technique, or, in the case of the novel, more expensive treatment, an additional funding mechanism.

To illustrate the concept of the proposed clinically-integrated randomized trial, and demonstrate how each of its four elements might be implemented in practice, we will describe its possible application in four distinct areas: surgery, "me too" drugs, rare diseases and lifestyle interventions.

### Example 1: Surgery for prostate cancer

The surgical literature is replete with non-randomized studies describing modifications to standard surgical procedures and the results thereof[5,6]. Randomized trials are much rarer. With respect to radical prostatectomy for prostate cancer, our focus here, we have only been able to find only a handful of trials, including a total of fewer than 1000 patients in total, that have compared different surgical approaches, such as a retropubic versus perineal approach[7] or bladder neck eversion versus vesico-urethral anastomosis without bladder neck eversion[8]. In contrast, simple searches on Medline find well over 2000 non-randomized studies on radical prostatectomy.

One possible reason why such trials are so rare is that the cost of such trials far outweighs the value of the information obtained. For example, imagine that a surgeon developed a technique which was hypothesized to decrease rates of impotence, a common side-effect of radical prostatectomy, by about 5%. A suitably powered trial might require some 3,000 patients. It is not unusual for a trial to cost upwards of $5,000 per patient [9-11], giving total costs of at least $15 m. It is implausible that any funding body would fund a $15 m trial to look for a 5% difference in potency rates after radical prostatectomy.

A clinically-integrated randomized trial in radical prostatectomy would take the following general form.

1. The patient is first informed about the research during initial consultations. He is informed that while a lot is known about the best way to conduct a radical prostatectomy, and although it is known that a radical prostatectomy improves survival, there is considerable doubt about some technical aspects of the procedure. Accordingly, the routine practice of the surgeon is to conduct the surgery in the way that is known to be best for the patient but to randomize certain other aspects of surgery about which there is uncertainty: the surgeon includes all, or nearly all, of his or her patients on the randomized trial, and some procedures are only available to trial patients.

2. All patients, whether or not they are on the study, complete a short questionnaire about their baseline urinary and erectile function using a web-interface. Data from the questionnaire is added directly to their medical record. Use of a web-based interface for patient-reported outcomes after cancer treatment has been shown to provide high-quality data[12].

3. If the patient consents to the study, his electronic medical record is flagged. The clinical database then communicates with a research database which undertakes randomization.

4. The trial may involve several different arms. For example, the trial may, at the same time, examine a novel technique thought to reduce the risk of shedding of cancer cells into the circulation and thus cancer recurrence; compare two approaches to nerve-sparing to determine effects on potency; examine whether preserving an anatomic feature can improve postoperative urinary function. The trial might also include randomization within sub-categories of procedure. For example, patients receiving a care from a surgeon who uses a laparoscopic approach might be randomized to one of two different laparoscopic devices.

5. Randomization is also stratified by surgeon, such that each surgeon will treat approximately equal numbers of patients with each technique. Randomization also uses a minimization ("biased coin") approach to ensure broad comparability of prognostic factors between groups[13]: this important because one surgeon may see only limited numbers of patients. Data on prognostic factors is obtained directly from the electronic medical record.

6. The results of randomization are sent directly to the electronic medical record with a copy to the surgeon by email. The electronic medical record would also record that the patient had been randomized. After surgery, the surgeon uses tick boxes on the electronic medical record to document the actual procedures used during the surgery.

7. Patients are sent emails at home every few months. This provides a link to a secure website containing an electronic questionnaire about urinary and erectile function. Patient's responses to the questionnaire are uploaded directly to the patient's electronic medical record. Patients without home Internet access can access the website using a hospital computer when they return for routine follow-up visits. Exactly the same emails are sent to patients who do not consent to the trial, as they are a standard part of clinical follow-up.

8. The study statistician can download data directly from patient's electronic medical record by running a query through the central study database. The query would automatically de-identify data so that patient privacy is protected. All pertinent endpoints should be directly available from the patient record including cancer characteristics, such as stage and grade; surgical details such as operating time, blood loss, length of stay, complications and surgical margins; oncologic results, such as cancer recurrence; functional outcomes, such as urinary and erectile function, obtained from the electronic questionnaires.

9. The trial can open and close arms independently. For example, a comparison of two devices, the primary endpoint of which is operating time, may require far fewer patients than a study with cancer recurrence as an endpoint. Similarly, an arm could close if a treatment was found to be harmful during the trial. Accordingly, the clinically-integrated trial would consist of a single master protocol, continuing over many years. This would drastically reduce the time and effort required to test new questions. This is not a trivial consideration: it has been estimated that opening a clinical trial through Cancer and Leukemia Group B, one of the main cooperative groups that conduct multicenter trials in cancer, typically requires 370 separate steps and about two years[14].

Note that the trial is of factorial design, that is, patients can receive one intervention, a different intervention, both or neither. This allows the study hypotheses to be addressed with far fewer patients. A factorial design is most appropriate when there is no interaction between treatment arms, that is, when the effects of one surgical modification, say to protect potency, are similar in patients with and without the other experimental modification, say to improve continence rates. We believe that this condition will be met for most surgical modifications.

Such a design might allow either patients or surgeons to "opt out" of particular comparisons. For example, if a surgeon felt that a particular procedure for preserving potency was associated with excess risk of recurrence, that surgeon's patients would always be assigned so that they did not receive that procedure; however, these patients could still be randomized with respect to other comparisons in the trial, such as techniques to improve continence.

### Example 2: Rare disease and "me too" drugs

Rare diseases and "me too" drugs paradoxically present the physician with a similar problem: choosing a therapy out of a bewildering array of possibilities. For rare diseases this is because difficulties of accruing patients to large trials mean that there are often only a few treatments that have been proven to be effective, leaving the physician to try unproven approaches for patients who do not respond; in the case of "me too" drugs, there are many treatments known to be effective, but often none has been proven superior to another.

To illustrate how a clinically-integrated randomized trial could be of benefit, take the case of a patient with newly diagnosed depression. The patient's doctor recommends antidepressant medication, and explains why this might help. The doctor then states that although all prescription antidepressants are known to work, exactly which is most effective is unknown, and proposes that the patient takes part in a randomized trial in which the drug is chosen at random. If the patient agrees, he or she would complete a depression questionnaire. The doctor, or office assistant, would then access a secure study website and enter a limited amount of basic patient data, which would include any co-morbidities, age and contact details, as well as the results of the depression questionnaire. The study database would then randomize the patient and present a "results screen" showing the allocated drug. This drug is then prescribed in the normal way, with the patient collecting the drug at the local pharmacy. The patient is then sent an email at 6 and 12 weeks with links to the depression questionnaire and a brief questionnaire about side-effects. The trial might also contact the patient at long-term follow-up, say 12 – 24 months, and include questions about depression, drug compliance and use of other therapies. The results of the questionnaire would be sent to the patient's doctor so that he or she could adjust the patient's care appropriately, for example, by changing treatment if response was poor (indeed, one advantage for doctors to participate in the trial might be that such a system might be made available to all their patients, whether or not they took part). Outcomes such as hospitalization or death – important in a comparison of say, different statin drugs – could be obtained by computerized linkage to national databases. This approach has been shown to feasible and accurate[15] and has been used in the analysis of a large randomized trial[16].

A similar schema could be used for the treatment of a rare disease: explanation to the patient that the most appropriate course of treatment is unknown; web-based completion of baseline data and randomization; treatment given by the doctor according to the results of randomization; follow-up via email. Such a trial could be conducted worldwide, involving all major centers where the rare disease is treated, although this would require worldwide agreement on which interventions to test.

### Example 3: Lifestyle interventions

There is a bewildering array of diets for weight loss, and the US National Institutes of Health has accordingly sponsored several major randomized trials. While no doubt providing valuable information, these trials have been relatively small and expensive (for example, 311[17] or 160[18] patients accrued at a cost of $1 – $2.5 m [data from authors]). A sample size of 300 is somewhat less of a drop in the bucket of the approximately 50 million Americans who attempt to lose weight each year[19]. A clinically-integrated randomized trial would allow patients to log on to a website, read appropriate information and warnings about the trial, take a short test to confirm their understanding, provide baseline information – such as age, weight, previous diet history and perhaps a psychological questionnaire – and then be randomized to one of a number of different weight loss programs. Compliance and weight changes would then be assessed by patient self-report; patients would also have the opportunity to enter other data, such as cholesterol or blood pressure, if these were assessed in routine care. A similar methodology could be used for smoking cessation, or exercise regimens.

### Incentives for doctors and patients

There are several incentives that could be offered to doctors to encourage them to take part in a clinically-integrated randomized trial. In the case of the surgery trial, where many of the trial doctors will be academics, an excellent incentive would be to allow all participants access to deidentified raw study data. This would allow these doctors to test any additional, secondary hypotheses of interest to their scientific work. In the case of the "me too" drug trials, and trials of rare diseases, many of the participating doctors will be in the community and offers of data sharing are unlikely to be highly valued. A modest payment, to compensate for the minor additional work involved randomizing a patient, might comprise a sufficient incentive for many doctors.

In addition, response adaptive allocation ("play-the-winner") might be considered[20]. This works as follows: let us imagine that the depression trial randomizes between four drugs: A, B, C and D. For the first 100 patients or so, each patient has an exact 25% chance of receiving any particular treatment. The results of the first 100 patients are then analyzed. Now assume that response rates were 50%, 60%, 50% and 40% for drug A, B, C and D respectively. The randomization scheme is then adjusted so that patients have a slightly higher chance (say 30%) of being randomized to drug B, which current data suggests is the most effective, and a slightly lower chance (say 20%) of being randomized to the currently least effective drug D. These randomization probabilities can be updated continuously as the trial progresses. Although the results are not made public, doctors could say to their patients, "I really don't know which is the best drug, nobody does, but if we put you on the trial, you have a higher chance of getting the most effective treatment – based on the experiences of all the patients that have been treated so far – compared to if I just take a guess and choose a treatment for you". Similarly, a trial comparing diets would undoubtedly be more attractive to patients if they had a higher chance of being randomized to the diet which had currently led to greatest weight loss. Response adaptive allocation can be inefficient compared to equal randomization, and some authors have claimed that it subverts equipoise, the ethical imperative behind randomization[21] (although other authors have argued the opposite[22]). Nonetheless, the technique could be considered as a way to encourage patient and doctor participation.

The use of web-based questionnaires may also be seen as a benefit to patients. Patients like to see how they are doing, and the web system could be programmed to provide feedback to patients as to their progress in an attractive and user friendly manner.

That said, the design of the clinically-integrated trial is such that important incentives may be unnecessary. We typically use incentives to get someone to do something they would not otherwise want to do, like undergo testing or fill in lengthy questionnaires. If patient care is essentially unaltered by the trial, the question of incentives becomes somewhat moot especially if randomization is routine for all patients.

### Barriers to clinically-integrated randomized trials

We see four potential barriers to adoption of clinically-integrated randomized trials: methodologic, technical, practical and regulatory. The methodologic issue is that

clinically-integrated randomized trials ask about the effectiveness of treatments in routine clinical practice. This is generally the domain of what are known as "pragmatic" trials, which cannot have the sort of rigorous controls associated with "explanatory" trials[23], such as blinding or careful outcome assessment (for example, in a diet trial, patients would weigh themselves). Instead, one would need to trust that there would not be important preferences – leading to bias – between different similar alternatives, and that the sort of large sample sizes possible with clinically-integrated trials – 10, 20 or 50 fold greater than with traditional trials – would offset any increase in variance associated with sub-optimal outcome assessment, or the possibility of cheating by a minority of participants with a vested interest in the results.

The technical issues are distinct for the surgery trial as compared to trials of "me too" drugs, rare diseases or lifestyle interventions. The surgery trial requires integration of the electronic medical record and the research database. This requires that all institutions taking part in the trial use either a similar form for the electronic medical record, or design a special interface between the institution's medical record system and that of the trial. In the case of prostate cancer, many institutions currently use an electronic medical record system known as CAISIS[24] that could easily be adapted for use in a clinically-integrated randomized trial. Institutions not using CAISIS would require special programming code to be written to allow communication between the research and clinical databases. This can be expensive, but would only need to be done once for each institution. For trials of "me too" drugs, rare diseases or lifestyle interventions, the trialists would need to establish a website with separate interfaces for physicians, patients and study investigators. Again, this might incur considerable costs, but those costs would only need to be paid once and would certainly not approach the many millions of dollars currently associated with large trials.

A practical limitation concerns patient access to the Internet. Yet Internet penetration is deep and growing – in 2007 about 61% of British households had Internet access, a proportion growing by 7% per year[25] – and telephone systems can be used to obtain symptoms for patients without Internet access: a recorded message asks, for example, for patients to rate their pain on a 0 – 6 scale, and the patient presses 0 – 6 on the telephone key pad as appropriate.

A limitation related to the Internet is whether patients might give biased or even maliciously false responses. Although we do not think that patient bias would be important for most clinically integrated trials – for example, we do not think patients would have a preference between different modifications of surgical technique – it is possible that trials of drugs, or of diets, might be subject to Internet abuse. For example, proponents of a diet might sign up to a trial and claim to have lost weight if randomized to their favorite diet and to have gained weight otherwise. We think that a limited number of simple steps could reduce the possibility of fraud. First, patients consenting through the web could be sent by mail to their home a code number to be entered the first time that outcome information is entered. This would prevent a single individual from submitting multiple "ghost" entries as the web system would easily recognize repeat addresses. Second, patients would be asked if they would be willing to be audited, which would involve face-to-face contact with a member of the study team. Although, a proportion of patients would indeed be audited, the primary purpose of the audit would be to explore any unusual patterns of those unwilling to be contacted further. That said, clinically-integrated randomized trials are not blinded, and so are prey to similar considerations of bias as other open label trials. If the investigators believe that the risk of bias from lack of blinding outweighs the benefits of large sample sizes, then a clinically-integrated randomized trial is not an appropriate methodology.

Perhaps the most significant barrier to clinically-integrated randomized trials is regulatory and concerns ethical oversight. Although in the surgery trial it would be feasible to obtain ethical approval from all participating centers, trials of "me too" drugs or, particularly, rare diseases, are likely to involve very large numbers of geographically diverse sites, many of which may enter only one or two patients. It is simply not feasible to attempt to obtain local ethical approval separately for all doctors who might enter patients in such a trial. The obvious solution would be for national or regional oversight entities to grant waivers to allow physicians to enter patients on trials without local ethical approval if the trial as a whole has been approved by a reputable review committee. Whether local ethical committees would be willing to relinquish oversight is another matter. Moreover, for a trial of a rare disease conducted globally, it might be complex to obtain ethical approval for all countries involved, even if local approval was not needed and only a single national approval required. In the case of lifestyle trials, where patients can enter themselves on study, matters of ethical oversight remain unclear: who is responsible for overseeing a trial where thousands of patients sign on to a trial via a website?

## Conclusion

The current proposal involves a novel way of thinking about and conducting clinical trials. To see why this is unusually innovative, we might consider what fundamental innovations have been made to randomized trials since their inception. There have been relatively few: cluster randomization has been developed to deal with interventions that can affect more than one patient at a time, and group sequential methods have allowed ethically appropriate (and scientifically sound) evaluation of interim results. Perhaps the most fundamental innovation to randomized trials that has become a common part of research practice is the "large, simple trial"[4]. The clinically-integrated randomized trial has much in common with the large, simple trial: moderate effects are clinically important; to detect such effects requires large trials; to be large, trials must be simple. However, the clinically-integrated randomized trial is distinct in its emphasis on making randomization part of routine clinical care and in the attempt to randomize every patient (this is analogous, but importantly different, to Chalmers' call to "randomize the first patient"[26]). Moreover, large, simple trials have generally been used to determine whether treatments work at all (e.g., fibrinolytics for heart attack) rather than to evaluate different modifications to a therapy of known effectiveness. The development of information technology is also central to the clinically-integrated randomized trial as it allows the incorporation of patient-reported outcomes; typical large, simple trials have used mortality as an endpoint.

Our proposal is neither a replacement for more traditional designs (see table 1) nor a "one-size-fits-all" methodology: many design decisions would need to be taken irrespective of whether trialists chose a clinically-integrated or more traditional design. Take, for example, whether only highly experienced surgeons would be eligible compared to all surgeons; which questionnaires would be given and when; and how crossover between different drugs would be handled statistically: such questions are not determined by the degree of clinical integration of the trial but by the specific research questions being addressed.

**Table 1:** Features of trials appropriate for clinically-integrated versus more traditional randomized trials.

	**Clinically-integrated randomized trial**	**Traditional randomized trial**
Tests, procedures, questionnaires	All data needed to address the study question would be of value for the clinician during routine follow-up. All tests, procedures and questionnaires would be given to patients irrespective of participation. *Example: A test for cancer recurrence*.	Some data required to answer study-specific questions would not be taken during routine care. *Example: blood draw for a molecular marker thought to predict response to treatment*.

Treatments	Patients very unlikely to have strong preferences for one or other treatment. *Example: Two alternative suturing techniques during surgery.*	Many patients may have a strong preference for one or other treatment. *Example: Radical versus breast conserving surgery.*

Comparisons	Can only compare two active treatments. *Example: Two widely used anti-depressants of proven value.*	May compare an active treatment to placebo or no treatment control. *Example: Novel anti-depressant versus placebo.*

Patients	Most patients are randomized.	Only a proportion of patients are randomized.

Eligibility criteria	Eligibility criteria should be minimized. *Example: all patients undergoing radical prostatectomy are eligible*.	Eligibility criteria can be restrictive. Example: *restrictions on comorbidities in a trial of a novel drug*.

Information technology	Trial depends heavily on information technology.	Trial can be lo-tech.

We believe that the randomized trial is the best method for determining the optimal treatment from comparable alternatives, be they modifications to surgery, "me too" drugs, treatments for rare diseases or diets for weight loss. Yet contemporary randomized trials have become increasingly expensive, complex and burdened by regulation, so much so that many trials are of doubtful feasibility. Our proposed clinically-integrated randomized trial is a streamlined approach to randomized trials that may allow us to enlarge dramatically the number of clinical questions that can be addressed by randomization. That said, our proposal is just that, a proposal: there has been no practical experience of the methodology and we do not know, amongst other things, whether doctors would agree to take part in clinically-integrated randomized trials, whether patients would consent to them, whether the trials would be approved by ethical bodies and whether the trials would indeed be low cost. We intend to experiment with the methodology in the near future by conducting a trial of modifications to radical prostatectomy.

## Competing interests

The authors declare that they have no competing interests.

## Authors' contributions

AV developed the idea for the study with the support of PS. Both authors read and approved the final manuscript.
